# First Cryo-Scanning Electron Microscopy Images and X-Ray Microanalyses of Mucoromycotinian Fine Root Endophytes in Vascular Plants

**DOI:** 10.3389/fmicb.2020.02018

**Published:** 2020-09-03

**Authors:** Felipe E. Albornoz, Patrick E. Hayes, Suzanne Orchard, Peta L. Clode, Nazanin K. Nazeri, Rachel J. Standish, Gary D. Bending, Sally Hilton, Megan H. Ryan

**Affiliations:** ^1^School of Agriculture and Environment, Institute of Agriculture, The University of Western Australia, Perth, WA, Australia; ^2^Centre for Microscopy, Characterisation and Analysis, The University of Western Australia, Perth, WA, Australia; ^3^School of Biological Sciences, The University of Western Australia, Perth, WA, Australia; ^4^Environmental and Conservation Sciences, College of Science, Health, Engineering and Education, Murdoch University, Murdoch, WA, Australia; ^5^School of Life Sciences, University of Warwick, Coventry, United Kingdom

**Keywords:** arbuscules, cryoSEM, *Glomus tenue*, Mucoromycotina, *Planticonsortium*

## Abstract

**Aims:**

Arbuscule-producing fine root endophytes (FRE) (previously incorrectly *Glomus tenue*) were recently placed within subphylum Mucoromycotina; the first report of arbuscules outside subphylum Glomeromycotina. Here, we aimed to estimate nutrient concentrations in plant and fungal structures of FRE and to test the utility of cryo-scanning electron microscopy (cryoSEM) for studying these fungi.

**Methods:**

We used replicated cryoSEM and X-ray microanalysis of heavily colonized roots of *Trifolium subterraneum*.

**Results:**

Intercellular hyphae and hyphae in developed arbuscules were consistently very thin; 1.35 ± 0.03 μm and 0.99 ± 0.03 μm in diameter, respectively (mean ± SE). Several intercellular hyphae were often adjacent to each other forming “hyphal ropes.” Developed arbuscules showed higher phosphorus concentrations than senesced arbuscules and non-colonized structures. Senesced arbuscules showed greatly elevated concentrations of calcium and magnesium.

**Conclusion:**

While uniformly thin hyphae and hyphal ropes are distinct features of FRE, the morphology of fully developed arbuscules, elevated phosphorus in fungal structures, and accumulation of calcium with loss of structural integrity in senesced arbuscules are similar to glomeromycotinian fungi. Thus, we provide evidence that FRE may respond to similar host-plant signals or that the host plant may employ a similar mechanism of association with FRE and AMF.

## Introduction

A recent study suggested that the arbuscule-producing fungi, fine root endophytes (FRE), belong to the subphylum Mucoromycotina, and not the Glomeromycotina (Orchard et al., 2017a). This finding was the first report of arbuscule-producing fungi outside of Glomeromycotina (formerly Glomeromycota; Spatafora et al., 2016), the subphylum within which FRE were placed previously (as *Glomus tenue*). Later, Desirò et al. (2017) proposed that FRE belonged in the Densosporaceae family within Endogonales, while Walker et al. (2018) proposed that the only described species of FRE, *Glomus tenue*, be placed in the new genus *Planticonsortium*. However, it is not yet clear if all species of FRE belong to a single genus. Hence, here we refer to them simply as FRE. Even though both glomeromycotinian arbuscular mycorrhizal fungi (AMF) and FRE are arbuscule-forming fungi, we use this terminology to differentiate those from Glomeromycotina and Mucoromycotina, respectively.

Researchers have recently regained interest in Mucoromycotina after the subphylum was suggested to aid the colonization of land by plants, together with Glomeromycotina (Bidartondo et al., 2011; Feijen et al., 2018; Hoysted et al., 2018, 2019). Moreover, from their comprehensive literature review, Orchard et al. (2017b) concluded that FRE are likely abundant within the roots of plants in both agricultural and natural ecosystems. Colonization by FRE has occasionally been suggested to aid host plants, with some similar benefits to colonization by AMF; for example, enhanced nutrient uptake, particularly of nitrogen (N) and phosphorus (P) (Crush, 1973; Orchard et al., 2016b; Hoysted et al., 2019). Recent research by Field et al. suggested that FRE might be a mutualistic symbiont, similar to AMF (Field et al., 2015, 2016, 2019). However, overall, while glomeromycotinian fungi (i.e., AMF) are well researched (Smith and Read, 2008), we have a very poor understanding of FRE and their interactions with the host plant. For example, even though it is likely that FRE conduct nutrient-for-carbon exchange through their arbuscules (similar to AMF), it remains unclear whether the mechanisms and processes of this exchange are shared between AMF and FRE.

Fine root endophytes have distinct morphological features within roots of vascular plants when stained, and their thin hyphae are easily distinguished from those of AMF at magnifications of ≥ 100 × (Thippayarugs et al., 1999). The definitive structure associated with FRE within the epidermis and outer cortex of the root is a fan-shaped or palmate entry point. Subsequently, FRE produce diverging fine hyphae (< 2 μm diameter) branching both inter- and intracellularly to spread within the root cortex (Greenall, 1963; Gianinazzi-Pearson et al., 1981; Abbott, 1982). The aseptate hyphae produce terminal and intercalary vesicle-like swellings and fine, delicate, arbuscules in the mid to inner cortex of the root (Gianinazzi-Pearson et al., 1981; Orchard et al., 2017a). When colonies of FRE and AMF intermingle, the arbuscules of each are difficult to discriminate in stained roots. Spores of FRE were reported to be ≤ 20 μm and colorless when young, turning dark brown with age (Hall, 1977; Brundrett et al., 1996), making them difficult to identify or isolate.

Since the 1970s, electron microscopy has allowed high magnification visualization of plant root inter- and intra-cellular structures and been used to elucidate the morphology of AMF. Transmission electron microscopy studies examined AMF *in planta* and described functional and developmental traits (e.g., Kinden and Brown, 1975b, c, 1976). For example, the lifecycle of AMF in roots and, particularly, the development and degradation of arbuscules from dichotomous branching of the trunk hypha to senesced arbuscule branchlets amassing into clumps (Kinden and Brown, 1976). Such studies paved the way to advance our understanding of the nature of the associations between host plants and AMF.

Cryo-scanning electron microscopy (cryoSEM) allows for the analysis of fungal structures and element concentrations within frozen-hydrated root cells, reporting them in their native state, and therefore avoiding any extraction artifacts that may occur with alternative solvent based preparations (McCully et al., 2009; Hayes et al., 2018). This methodology has become increasingly popular for studies of nutrient distribution in plant tissue, such as leaves (Marshall and Xu, 1998; McCully et al., 2010; Jin et al., 2017; Ding et al., 2020). However, it has been used only a handful of times to describe intraradical cell structures of plant roots in symbiosis with AMF (e.g., Ryan et al., 2003, 2007). For example, Ryan et al. (2003) made precise measurements of AM fungal structures and classified AM fungal arbuscules into three developmental stages: young, collapsing and clumped. This finding was consistent with the earlier observations of Kinden and Brown (1975c, 1976). When combined with X-ray microanalysis to measure elemental concentrations, cryoSEM has advanced our understanding of host–fungal nutrient transfer and allocation. For instance, intraradical hyphae contained high concentrations of P (60–170 mM) balanced by potassium (K) and, to a lesser extent, magnesium (Mg) (Ryan et al., 2003, 2007). These elemental concentrations were reduced in the arbuscules, especially that of P (30–50 mM), consistent with the elements being transported to the plant (Ryan et al., 2003). In the same study, highly elevated calcium (Ca) concentrations (30–250 mM) were measured in collapsing and, especially, senesced “clumped” arbuscules. In contrast to the knowledge of AM fungal structures, biochemical descriptions and analyses of FRE have been largely unexplored since Gianinazzi-Pearson et al. (1981) published their study of observations of chemically fixed (in 3.5% glutaraldehyde) roots under SEM.

Several attempts at describing the morphology of FRE have been reported (Walker and Powell, 1979; Gianinazzi-Pearson et al., 1981; Field et al., 2015, 2016, 2019). However, these studies did not use cryoSEM, were conducted on early divergent non-vascular plants, or did not link morphology to elemental concentrations. Walker and Powell (1979), however, used X-ray microanalyses to measure nutrients in arbuscules but did not differentiate between FRE and AMF structures. Nevertheless, Field et al. (2015; 2016; 2019) and Hoysted et al. (2019) provided SEM images and evidence of carbon-for-nutrient transfer from arbuscules of FRE to the rhizoids of liverworts and an early divergent vascular plant. Hence, there is a need for a systematic and replicated study to accurately evaluate the morphology and elemental concentrations within the structures of FRE in plants that form true mycorrhizal associations (i.e., vascular plants).

Even though studies are limited, biochemical differences have been noted between FRE and AMF. For example, the ultra-cytochemical staining of the hyphal walls of FRE showed two distinct layers, while only one was visible in AMF, suggesting differences in polysaccharide content (Gianinazzi-Pearson et al., 1981). Further, during arbuscule senescence, no septa developed as previously observed for AMF (Cox and Sanders, 1974; Kinden and Brown, 1975b), and biochemical processes differed slightly during fungal wall aggregation with no indication that the host plant assimilated the degrading structures of FRE, as hypothesized with AMF (Gianinazzi-Pearson et al., 1981). Hence, these earlier findings suggest that some biochemical processes within FRE may differ from those in AMF and that further analysis of the differences will advance the research of FRE and understanding of their potentially mutualistic roles.

In this paper, we present the results of cryoSEM analyses coupled with X-ray microanalysis of *Trifolium subterraneum* roots colonized by FRE. *Trifolium subterraneum* is one of the most successful exotic annual pasture legumes in southern Australia (Nichols et al., 2012) and, hence, of economic importance. Here, we aimed to characterize the inter- and intracellular structures of FRE through cryoSEM and estimate nutrient concentrations in plant and fungal structures using X-ray microanalysis. Further, we aimed to identify defining *in planta* features of mucoromycotinian FRE that could allow differentiation from glomeromycotinian AMF going forward.

## Materials and Methods

Plants highly colonized by FRE and non-colonized by AMF were produced in two sequential glasshouse experiments. First, we wet-sieved soils from a pasture known to be enriched in FRE through a series of sieves and retained the material on the 50 μm sieve (as for Experiment 1 in Orchard et al., 2017a). Material obtained from the 50 μm sievings contained few or no propagules of AMF but had retained spores of FRE (McGee, 1989; Thippayarugs et al., 1999). We then added this material to pasteurized soil and conducted a dilution series to produce a final dilution of 1: 81 and 1: 162. Twenty pots per dilution were sown with two *T. subterraneum* plants. Plants were grown for 8 weeks in a glasshouse. No additional nutrients were applied. Pots were free-draining and watered as required. At harvest, each plant was carefully dug up and removed from the pot; soil adhering to roots was shaken back into the pot. Each root system was washed with tap water and excised from shoots. Roots were stored in 70% ethanol and later stained using the vinegar-ink method (Vierheilig et al., 1998) in preparation for the visual assessment of FRE and AMF, if any (see method below). The remaining soil in each pot was air-dried and stored. Many of the 20 pots from the 1: 162 dilution showed no colonization by AMF, but high colonization by FRE. Soil from these pots was used in a second experiment. Other dilutions contained colonization by AMF and hence, were not used further.

In the second experiment, all soil from each of the pots containing only FRE was mixed with pasteurized, washed, coarse river sand (1: 3), placed into five large pots, and two *T. subterraneum* plants grown. Each pot was enclosed in a clear bag; no additional nutrients were applied. Pots were closed and watered to 80% field capacity weekly. After 10 weeks, plants were harvested. Roots from each plant were washed with tap water and excised from shoots. Excess moisture was absorbed by a paper towel, and the root system laid out with the roots aligned. We avoided using root zones within 20 mm of the apex or tip to give root pieces of a consistent diameter. Three subsamples of the aligned roots were taken: one for cryoSEM (see below), one for DNA analysis, and one for visual assessment of colonization.

### Roots for Visual Assessment

Roots were rinsed in tap water and placed into 10% KOH for clearing, and then stained with ink and vinegar (Vierheilig et al., 1998; Walker, 2005). Colonization was scored by examining at least 30 approximately 15 mm root pieces mounted onto microscope slides and examined at magnifications of 100× and 400×, at a minimum of 100 intercepts using a modified version of the magnified intercept method (McGonigle et al., 1990). This method gave the percentage of root length colonized by FRE and AMF (Orchard et al., 2016a). Colonization by AMF was not detected (i.e., no thick hyphae, vesicles or arbuscules typical of AMF). Four pots were chosen to be used in the cryoSEM study because they had high colonization by FRE.

### DNA Sequencing and Analyses

DNA was extracted using FastDNA SPIN Kit for Soil (MP Biomedicals LLC, United Kingdom), according to the manufacturer’s instructions, with the exception that samples were homogenized in a Mini Beadbeater-8 cell disrupter for 3 min (Biospec Products, Inc., United States). A region of the 18S rRNA gene (*c*. 260 bp) was amplified from 15 ng DNA using the AMF fungal primer pair AMV4.5NF (5′-AAGCTCGTAGTTGAATTTCG-3′) and AMDGR (5′-CCCAACTATCCCTATTAATCAT-3′) (Sato et al., 2005). This primer pair targets a broad range of Glomeromycota as well as other fungal groups including the Mucoromycotina. PCR reactions were performed in a reaction volume of 25 μl, containing Q5^®^ Hot Start High-Fidelity 2X Master Mix (New England Biolabs) and 0.5 μM of each primer. Thermocycling consisted of an initial denaturation at 98°C for 30 s followed by 35 cycles of 98°C for 10 s, 60°C for 15 s and 72°C for 20 s. The final extension was at 72°C for 5 min. Following PCR, the DNA amplicon was purified using Agencourt AMPure XP beads (Beckman Coulter, United States) according to the manufacturer’s instructions. The adapted amplicon was then modified by attaching indices and Illumina sequencing adapters using the Nextera XT Index Kit v2 by PCR as described in the manufacturer’s protocol. Following the index PCR, the DNA amplicon was purified and normalized using the SequalPrep^TM^ Normalization Plate (96) Kit (Invitrogen) and then quantitatively assessed using a Qubit 2.0 Fluorometer (Life Technologies, United States). The final concentration of the library was 4 nM. The library was sequenced using the MiSeq Reagent Kit v3 600-cycle (Illumina).

Microbiome bioinformatics were performed with QIIME 2 2019.4 (Bolyen et al., 2018). Primers were trimmed from the reads using the cutadapt plugin (Martin, 2011). The DADA2 plugin (Callahan et al., 2016) was then used to filter the reads (reads were truncated at 210 nt based on a reduction in quality below a median of approximately Q30) and then join the forward and reverse reads, denoise and remove chimeras and singletons to produce ASVs (Amplicon Sequence Variants). Taxonomy was assigned at 99% using the Silva 132 SSU reference database (Quast et al., 2013).

### CryoSEM of Roots

The subsample of roots harvested from each pot were split into three to five small bundles, each containing ∼6 roots, of ∼5 mm in length. These were then gently bundled together, and each bundle placed vertically onto an aluminum pin using OCT^®^ and immediately cryo-preserved by plunging directly into liquid N, thereby immobilizing cellular ions. These were then stored in liquid N. After staining had identified four pots with high colonization by FRE and no colonization by AMF (see above), two to three cryo-preserved root bundles from each FRE colonized pot, with clearly identifiable hyphae and arbuscules, were selected and cryo-planned for imaging and elemental analysis (see below). Data for individual structures were then extracted from elemental maps, acquired from 16 individual roots, of the five plants with the highest amount of colonization by FRE and no colonization nor DNA sequences from AMF.

To prepare for cryoSEM imaging and X-ray microanalysis, each root bundle was cryo-planed in a cryomicrotome (Leica EM FC6 cryochamber integrated with Leica Ultracut EM UC6 microtome) at –120°C to produce a flat, transverse surface through the roots. To achieve this, roots were progressively microtomed using a glass knife at 1 μm, 750 and 500 nm steps, followed by a diamond knife at 250 and 100 nm steps. The planed samples were then transferred to a Leica MED020 cryopreparation system and sublimated at –100°C at 10^–7^ mbar (time determined empirically to remove upper surface water only), revealing the root structure and thereby enabling clear identification and visualization of plant and fungal structures (Supplementary Figure S1). This controlled sublimation has minimal effect on element concentrations (Marshall, 2017). Following sublimation, the samples were sputter-coated with 8 nm Cr and transferred to a Zeiss field emission SEM under vacuum for cryoSEM imaging. All imaging was done at 3 kV using the in-lens detector. After imaging, the samples were returned to the cyropreparation system, where an additional coat was added, for a total of up to 30 nm Cr. Element maps (energy dispersive spectroscopy, EDS) were then acquired at –150°C, 15 kV, and 2 nA beam current, a resolution of 512 pixels, and for > 4000 frames using an 80 mm SDD X-ray detector interfaced to Oxford Instruments AZtecEnergy software. Analyses were conducted, and quantitative data extracted from specific regions of interest as detailed extensively in Hayes et al. (2018) (also see Supplementary Figure S1). Details about quantification methods using SEM-EDS are described in detail elsewhere (Goldstein et al., 2018). In brief, regions of interest were drawn on the element maps and individual spectra from each pixel within the region of interest were summed and processed to yield element concentration data. Summed spectra from regions of interest were then quantified using the AZtecEnergy software. Percentage hydrogen (H) and N were fixed at 11.11 and 3.3%, respectively, with oxygen (O) determined by difference. All reported concentrations were total element concentration per kg wet weight, from fully hydrated root samples. This method of sample preparation and the fully integrated Oxford analytical system are highly suitable for elemental analysis and quantitation of biological samples (Huang et al., 1994; Marshall and Xu, 1998; Marshall and Clode, 2009; McCully et al., 2010; Marshall et al., 2012; Jin et al., 2017; Marshall, 2017; Hayes et al., 2018; Ondrasek et al., 2019). Due to the relatively low element concentrations in these plant materials, the element maps do not clearly visualize cellular differences across cellular regions, and thus are not included in the manuscript. However, quantitation of data from these analyses (calculated using spectra from regions identified on the map) is possible and, provides accurate concentration data even though these concentration variations are not visually evident in the element map (see Supplementary Figure S1).

Data were collected from four distinct cellular structures in each of the 16 root transverse sections examined, and bulked: (1) Developed arbuscules (DA; *n* = 33). Data were extracted from the area within a cell occupied by arbuscular hyphae, where the branches were clearly defined and not clumped. Due to the irregular shape of the arbuscules, this area unavoidably included some non-colonized parts of the cell, likely cell vacuole and cytoplasm and associated membranes; (2) Senesced arbuscules (SA; *n* = 45). Data were extracted from within the area of arbuscules that had clumped and where hyphae had lost their individual integrity; (3) Colonized cells, including vacuole and cytoplasm, avoiding areas with fungal structures (VC; *n* = 36); (4) Non-colonized cells, including vacuole and cytoplasm, only cells where no hyphae were present (NC; *n* = 176). Due to the small area of individual intercellular hyphae, no elemental data was collected from these structures. We also measured the hyphal width of both individual intercellular hyphae (*n* = 95) and hyphae that formed part of developed arbuscules (*n* = 118). The calculation of hyphal widths was performed using images resulting from the cryoSEM and the measuring software Klonk (Image Measurement Corporation, United States).

### Statistical Analyses

To evaluate statistical differences among hyphal widths and nutrient concentrations, we built linear mixed-effects models using plant as a random effect, since several roots were measured per plant (Pinheiro and Bates, 2000). The best model was then selected using Akaike information criterion and likelihood ratio tests (Zuur et al., 2009). Finally, residuals were visually inspected for assumptions of normality and homoscedasticity. Differences among cellular structures were identified using *post hoc* Tukey tests. To calculate the probability of finding hyphal bundles, we conducted a binomial generalized linear model with the “probit” link. The probability was then tested against the null model of a random expectation (i.e., 50:50 chance of finding single hyphae or a bundle of hyphae). Statistical analyses and graphs were made using the R statistical software package (R Core Team, 2016) with the “nlme” (Pinheiro et al., 2016) and “ggplot” (Wickham, 2016) packages, respectively.

## Results

### Colonization Levels and Diversity

**FIGURE 1:**
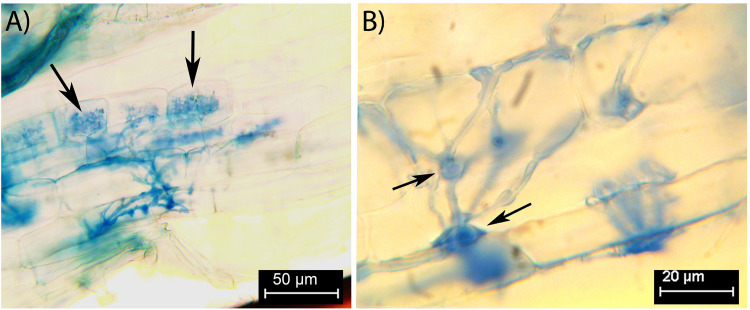
Fine root endophytes within stained *Trifolium subterraneum* roots, showing distinctive colonization patterns and structures. **(A)** Fine diverging hyphae and finely branching arbuscule (black arrows) in the inner cortex. **(B)** Intercalary vesicle-like swellings act as nodes close to an entry point in the outer cortex (black arrows).

Colonization by FRE was 68 ± 6% of root length (mean ± SE) for the five stained root systems, which were sourced from the same pots as the 16 roots used for cryoSEM and X-ray microanalysis. The colonization morphology was typical of that of FRE: darkly stained with fine, fan-like branching hyphae and plenty of finely-branched arbuscules (Figure 1A). Vesicle-like swellings were small (<13 μm), and often intercalary, acting as nodes in hyphal branching (Figure 1B). However, terminal vesicle-like swellings were also observed. There was no colonization by AMF in any root system.

From the DNA sequencing, we obtained 3546 fungal reads belonging to nine OTUs, six of which, comprising 99% of the reads, were assigned to the Endogonales (i.e., FRE) (Supplementary Table S1). The most abundant Endogonales OTU comprised 87% of the reads. We found no Glomeromycotina sequences (i.e., AMF) in our samples. This confirms that no AMF remained in samples after our sieving and dilution protocol. Five out of the six OTUs, including the most abundant one, matched the same reference sequence of FRE (Supplementary Table S1). Identity similarity for these OTUs ranged between 100% for the most abundant OTU, to 89% for the least abundant one (Supplementary Table S1).

### CryoSEM: Plant Intracellular Root Structures

**FIGURE 2:**
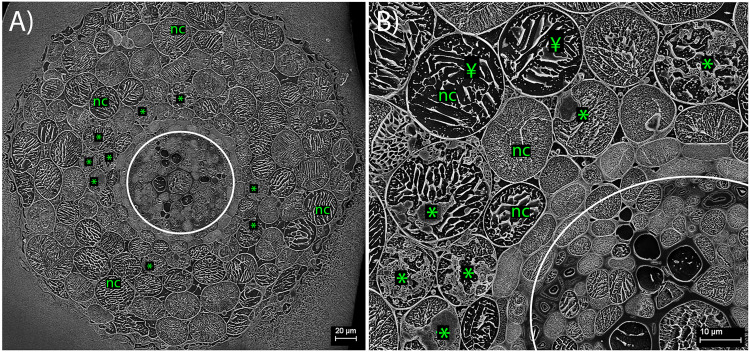
Cryo-scanning electron micrographs of *Trifolium subterraneum* roots colonized by fine root endophyte (FRE) in transverse section. **(A)** Full transverse profile showing the vascular tissue (within white circle), non-colonized outer cells (nc) and cells colonized by FRE in the inner cortex (*). **(B)** Higher magnification of the upper-left quarter of **(A)**. In non-colonized cells, the cell vacuole occupies nearly all the area within the cell wall (¥).

**FIGURE 3:**
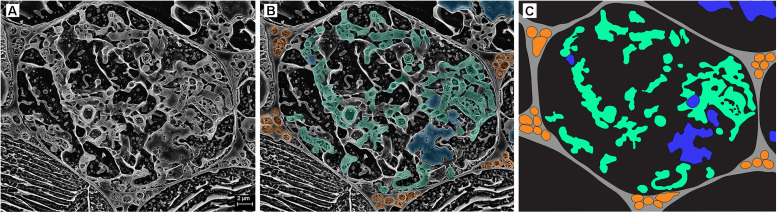
Cryo-scanning electron micrographs of a single cell within a *Trifolium subterraneum* root colonized by fine root endophytes in transverse section. **(A)** A colonized root cell containing an arbuscule entering senescence, showing a mixture of developed and senesced arbuscule branches. Image also shows ropes of three or more hyphae in every surrounding triangular-shaped intercellular space. The two neighboring cells on the bottom left and right show lines of sequestered solutes within the cell vacuole. **(B)** The same micrograph, now with key fungal structures highlighted in color: intercellular hyphal ropes (orange), developed arbuscule branches (green), and senescing arbuscule branches (blue). **(C)** Anatomical schematic showing only the structures of interest where gray and black represent intercellular and intracellular space, respectively.

Within the cryo-planed, sublimated, transverse sections of roots, the vascular tissue was discernible within the stele of the root (highlighted by the white circle in Figure 2), with associated large xylem vessels. Also, often visible around the outer root perimeter were collapsing, senesced epidermal cells, bound by the frozen OCT matrix (Figure 2A). In most cases, the vacuole occupied most of the planed cell area. Plant cells colonized by FRE were typically within the inner cortex, in a band 1–2 cells wide, and often contained arbuscules (Figure 2). Triangular intercellular spaces often contained intercellular hyphae of FRE, either individually or in bundles. Cell walls of the hyphae within the bundles appeared to be fused, forming one structure (Figures 3, 5B). The probability of observing a bundle of two or more hyphae was higher than the random expectation (χ^2^ = 6.72; *P* < 0.01).

### CryoSEM: Hyphae and Arbuscules

**FIGURE 4:**
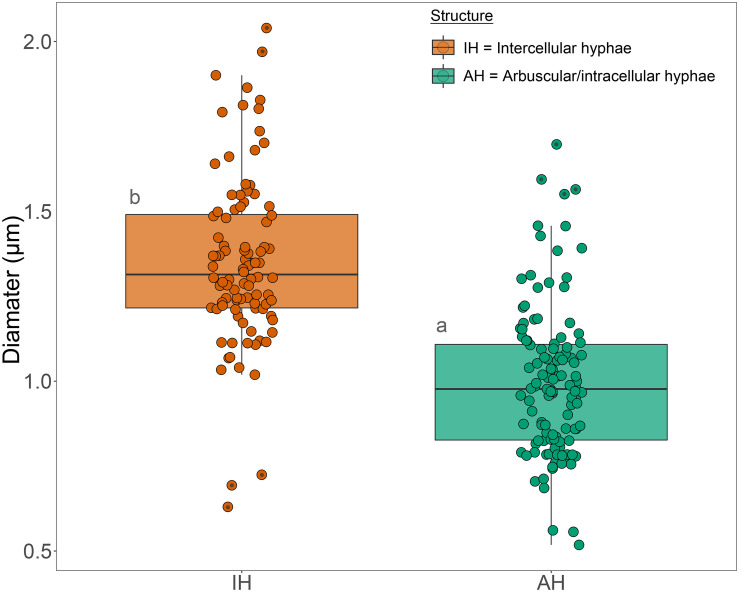
Box plot of the diameter measurements of intercellular hyphae and hyphae of developed arbuscules of fine root endophytes in the inner cortex of roots of *Trifolium subterraneum*. The box plots show the median, 90th percentiles and outliers (black dots). Different letters indicate significant (*P* ≤ 0.05) differences among treatment means based on *post hoc* Tukey tests.

In the inner cortex, intercellular hyphae often resembled rope-like structures, adjacent to cells with arbuscules, and presented a circular shape in the transverse plane (Figure 3). We refer to these bundles of hyphae as “ropes.” Hyphae varied in outer diameter between intercellular hyphae and developed arbuscules, with the former ∼36% wider than the latter (Figure 4). Hyphal diameter did not increase when the hyphae were in large intercellular spaces. Hyphae did not distort the plant cell wall when adjacent to it (Figure 3). Hyphal internal structure or contents were not discernible in the majority of the cryoSEM images or of sufficient area from which to extract element data.

**FIGURE 5:**
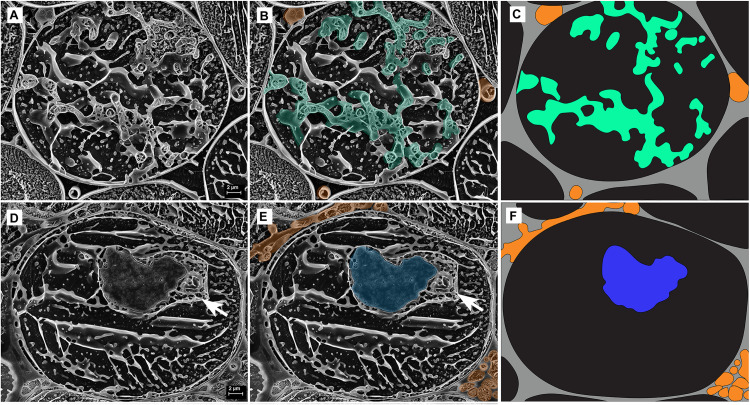
Cryo-scanning electron micrographs of two single cells from the inner cortex of a root of *Trifolium subterraneum* colonized by fine root endophytes in transverse section showing arbuscules at two developmental stages: **(A–C)** Developed arbuscule (green) and **(D–F)** senesced arbuscule (blue). The white arrow shows a structure often found adjacent to, and surrounding, senesced arbuscules. Intercellular hyphae are also shown (orange). **(C,F)** Anatomical schematic showing only the structures of interest where gray and black represent intercellular and intracellular space, respectively.

**FIGURE 6:**
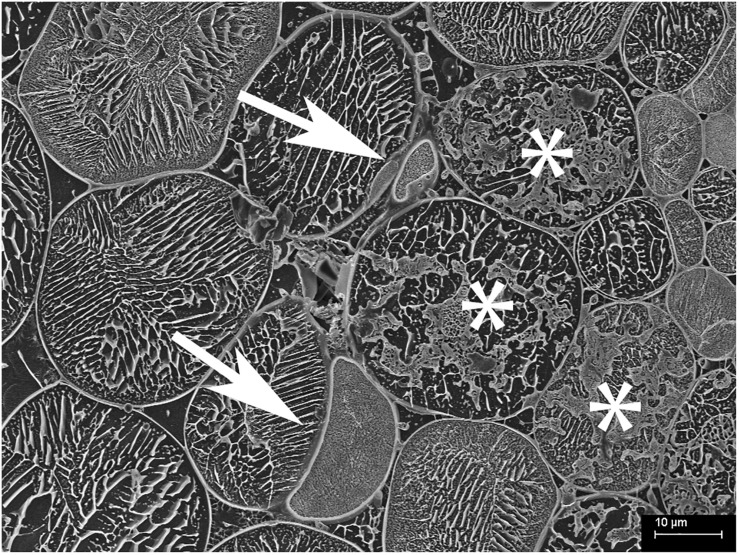
Cryo-scanning electron micrograph of cells from the inner cortex of a root of *Trifolium subterraneum* colonized by fine root endophytes in transverse section showing possible vesicle-like swellings in intercellular spaces (white arrows). The top vesicle-like swelling is attached to thin hyphae and surrounding colonized cells (*).

Arbuscules within the inner cortex were visible at varying stages of development (young through to senesced) (Figure 5). Developed arbuscules had turgid branchlets, ranging from 0.6–1.8 μm, surrounded by plant cytoplasm and the vacuole containing sequestered solutes (Figures 3, 5A–C). As the arbuscule senesced and reduced in size, a loss of definition was observed as hyphae coalesced into homogenous clumps (Figures 5D–F). Clumps were surrounded by a membrane, separating them from the cytoplasm. Often, a structure was seen immediately next to a senesced clumped arbuscule (Figures 5D,E). This structure was also surrounded by a membrane, separating it from the cytoplasm. In one instance only, structures consistent with being vesicle-like swellings were seen in intercellular spaces (Figure 6).

### X-Ray Microanalysis

**FIGURE 7:**
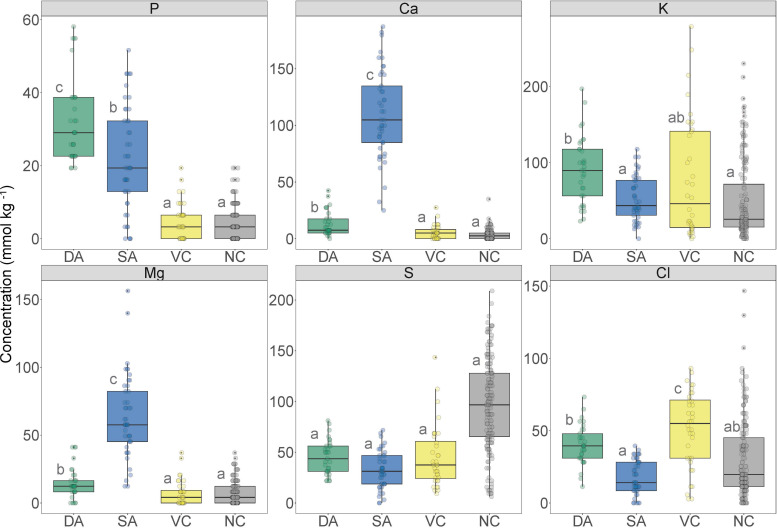
Boxplot of the nutrient concentration data obtained from X-ray microanalysis and extracted from defined regions of interest in transverse sections of *Trifolium subterraneum* roots colonized in the inner cortex by fine root endophytes. These data were obtained from defined regions containing developed arbuscules (DA; green), senesced arbuscules (SA; blue), vacuole and cytoplasm of colonized cells (VC; yellow) and vacuole and cytoplasm of non-colonized cells (NC; gray). Different letters indicate significant (*P* ≤ 0.05) differences among treatment means based on *post hoc* Tukey tests.

Quantitative elemental analyses were undertaken on the same samples from which correlative images were acquired. Data were only extracted from structures that were well-defined and readily recognizable in replicate samples. The data revealed elevated [P] within fungal structures [developed (DA) and senesced arbuscules (SA)] compared with non-colonized structures [vacuole and cytoplasm of colonized cells (VC) and non-colonized cells (NC)] (Figure 7). Phosphorus concentration in developed arbuscules was 32 ± 2 mmol kg^–1^ (mean ± SE), while in senesced arbuscules, the vacuole and cytoplasm of colonized cells, and non-colonized cells [P] was 22 ± 2, 5 ± 1 and 4 ± 1 mmol kg^–1^ (mean ± SE), respectively.

Concentrations of Ca were higher in fungal structures than non-colonized structures (Figure 7). Senesced arbuscules showed greatly elevated [Ca] at 109 ± 6 mmol kg^–1^ (mean ± SE), while developed arbuscules showed 13 ± 2 mmol kg^–1^ (mean ± SE). In contrast, the vacuole and cytoplasm of colonized and non-colonized cells showed very low [Ca] at 6 ± 1 and 3 ± 0 mmol kg^–1^, respectively.

Potassium concentration was highest in developed arbuscules and lowest in senesced arbuscules and non-colonized structures (Figure 7). Developed arbuscules showed a [K] of 89 ± 12 mmol kg^–1^ (mean ± SE), while senesced arbuscules, vacuole and cytoplasm of colonized and non-colonized cells had [K] of 47 ± 12, 66 ± 15, and 45 ± 12 mmol kg^–1^ (mean ± SE), respectively. Magnesium concentrations followed similar trends to [Ca], being highest in senesced arbuscules and lowest in non-colonized structures (Figure 7). Sulfur concentrations were not different among structures, while chlorine followed a similar trend to [K], except with the vacuole and cytoplasm of colonized cells showing the highest [Cl] (Figure 7).

## Discussion

The cryoSEM images revealed key morphological structures, particularly arbuscules, of FRE – the only known arbuscule-producing fungi outside Glomeromycotina. Single hyphae never occupied the entire intercellular space, as AMF tend to do (Ryan et al., 2003), but rather appeared closely fused within hyphal ropes. This seems to be a distinct characteristic of FRE. In contrast, X-ray microanalysis revealed similar patterns of nutrient concentrations to those of AMF, particularly during arbuscule senescence. These results suggest there are subtle but nonetheless clear morphological differences between FRE and AMF, but that the host-plant biochemical response to FRE during the arbuscule lifecycle may be similar to that of Glomeromycotinian AMF. We also show that communities of FRE colonizing *T. subterraneum*, are dominated by a single OTU, the same OTU dominating roots of *T. subterraneum* found in other studies (Orchard et al., 2017a). This further suggests that FRE, albeit being a group of species, are not very diverse within a single host.

### Morphology of Fungal and Plant Structures

Colonization morphology of the species of FRE evaluated here are in accordance with that described for the *Planticonsortium* genus (Walker et al., 2018). There appeared to be a distinct zone of arbuscular colonization by FRE, with arbuscules only observed in the inner cortex. Gianinazzi-Pearson et al. (1981) also noted this distribution. In contrast, AMF consistently colonize the outer, middle and inner cortex (Abbott, 1982; Yamato et al., 2011) or only the mid- to inner cortex (Ryan et al., 2003, 2007). Orchard et al. (2017b) also found FRE to colonize the mid- to inner cortex. Within and around colonized cells, the arbuscular branchlets of FRE were narrower than the intercellular hyphae, similar to that found for AMF (Ryan et al., 2003). However, the intercellular and arbuscular hyphal diameters of FRE were generally smaller (∼1.3 μm) than those of AMF (2–7 μm) as reported by Ryan et al. (2003) in *T. subterraneum*. Furthermore, single AM fungal hyphae often expand to fill the host intercellular space and even distort the wall of surrounding cells inwards (Ryan et al., 2003). In contrast, in our study, the intercellular hyphae of FRE were quite uniform in size and did not expand to fill the intercellular space, but rather multiple thin hyphae were closely grouped into hyphal ropes in the intercellular spaces, a trait also observed by Greenall (1963) and Gianinazzi-Pearson et al. (1981). Furthermore, even when these ropes occupied the entire intercellular space, they did not distort the adjacent host cell walls. The probability of finding intercellular hyphae in ropes exceeded a random expectation (i.e., 50:50 chance of finding a single hyphae or a bundle of hyphae) and, hence, it is likely that these hyphal ropes have a functional role. However, the presence of frequent hyphal ropes must have physiological implications; for example, a much greater abundance of cell walls and membranes per unit of hyphal volume for FRE relative to AMF would result in a greater requirement for phosphorus and carbon by FRE.

Only in one cryoSEM image did we find structures likely to be a vesicle-like swelling (Figure 6). The scarcity of these structures in the cryoSEM images is likely due to the low probability of a transverse cutting where a vesicle is present as they occur relatively infrequently compared with hyphae and arbuscules. The structures were of expected diameter, occurred close to colonized cells and appeared attached to the intercellular hyphae of FRE. Overall, our understanding of the function of the vesicles of FRE and the development of the spores of FRE is poor. In this study, we frequently observed vesicle-like swellings often in “chain of beads” structures under the optical microscope (Figure 1B), which differ from the general terminal type of vesicle formed by AMF (L. Abbott, unpublished data). This intercalary type of vesicle has been found in *T. subterraneum* roots on several morphotypes of FRE (Thippayarugs et al., 1999). Further research on the function and development of the vesicles of FRE is required; this may reveal additional biological differences between FRE and AMF.

### Fungal Nutrient Concentrations

There was an increased [P] in developed arbuscules of FRE compared with senesced arbuscules and non-colonized structures, which may reflect elevated P within the arbuscule branchlets and in their cell walls. Senesced clumped arbuscules also showed a significantly higher [P] than the non-colonized structures, and a structure (we speculate to be the host nucleus) was often associated with them. In cells colonized by AMF, Kinden and Brown (1975a, c) found an increase of mitochondria and ribosomes, paired with the occurrence of the host nucleus adjacent to clumped arbuscules. The authors attributed this to an increase in metabolic activity and hypothesized that nutrient transfer from fungi to host not only occurred in developed arbuscules, but also during the degradation of senesced arbuscules. However, further investigation is required to assess if the structure we often observed close to senescing arbuscules is indeed the nucleus. Previous studies of non-vascular plants (e.g., Field et al., 2019) provide clear evidence of P transfer from FRE to host plant, while one study on *Lolium perenne* showed physiological benefits in FRE colonization under P limitation (Crush, 1973). In addition, Ryan et al. (2003) showed the same elemental patterns in colonized roots for AMF, a well-known fungal group that enhances P uptake by their hosts. Overall, these studies in combination with our results suggest that FRE could be involved in P nutrition in vascular plants, similar to AMF, and might use similar nutrient-exchange mechanisms. This interpretation is further corroborated in a study by Hoysted et al. (2019) where they show P transfer from FRE to an early divergent vascular plant.

The concentration of P in the vacuole/cytoplasm of colonized cells was similar to that found in non-colonized cells. This contrasts with Ryan et al. (2003), who found vacuoles of colonized cells by AMF with a slightly higher [P] than non-colonized cells. In our study, plants were grown in sterile river sand with no nutrient addition to maximize mutualistic root colonization, while Ryan et al. (2003) grew plants in field soils with higher available P. When P soil availability is low, P is not stored in vacuoles as it is rapidly used by the plant (Lee and Ratcliffe, 1993).

There was a clear difference in [Ca] between non-colonized structures (i.e., vacuole and cytoplasm of colonized and non-colonized cells) and the area of cells containing either developed or senesced arbuscules of FRE. In fact, [Ca] in senesced clumped arbuscules was ten times higher than in developed arbuscules and a hundred times higher than in non-colonized cells. Increased [Ca] has been observed in the clumps that form during the senescence of AM fungal arbuscules, with concentrations of up to 65 mM measured; significantly greater than for non-colonized cell vacuoles of *T. subterraneum* (<10 mM Ca) and cells containing healthy arbuscules (26 mM) (Ryan et al., 2003). In this comparable study, the increased [Ca] at AM fungal arbuscule collapse was proposed to be induced by the host plant to initiate arbuscule senescence by these authors. However, no study has since attempted to evaluate Ca accumulation in senescing arbuscules. Ryan et al. (2003) hypothesized that this accumulation of Ca might be a byproduct of the termination of symbiosis between AMF and host plant. Therefore, the accumulation of Ca during senescence of the arbuscules of FRE hints at similar underlying biochemical processes controlling the lifecycle of FRE within the host root to those of AMF. Similarly, Mg followed the same trend as Ca. Magnesium availability has been shown to partially drive root colonization by AMF (Jentschke et al., 2000; Xiao et al., 2014). However, the cytological nature of this relationship is still unclear. Hence, the role of Ca and Mg in arbuscule senescing requires further investigation.

There was increased [K] within the area of cells containing developed arbuscules of FRE compared with senesced arbuscules and non-colonized cells. Potassium is essential for plant function, being involved in many processes including transport, enzymatic activity and, particularly, turgor pressure (Garcia and Zimmermann, 2014). Therefore, increased [K] in cells with arbuscules was likely due to the turgor activity in active arbuscule branchlets. This inference is supported by the reduction in [K] in cells containing senesced arbuscules: a similar trend was observed for AMF (Ryan et al., 2003). It was not possible to measure N due to practical issues. However, recent studies have suggested that endophytic mucoromycotinian fungi transfer N from soil to their early-divergent plant host (Field et al., 2015; Hoysted et al., 2019), and this requires further research to evaluate whether this is true to vascular plants. Previous studies have shown that arbuscular mycorrhizal inoculation can reduce plant S concentration (Taylor et al., 2015). In our study, we found no statistical differences in S among structures, but there was a trend where non-colonized cells showed higher S content than colonized cells. These data suggests there is no S transfer from fungus to plant. Finally, higher Cl content in developed arbuscules and vacuoles of colonized cells is most likely due to its involvement in turgor and osmoregulation (White and Broadley, 2001)

### CryoSEM to Evaluate Symbiosis

The method of sample preparation and the fully integrated Oxford analytical system we used in our cryoSEM approach are highly suitable for elemental analysis and quantitation of biological samples (Huang et al., 1994; Marshall and Xu, 1998; Marshall and Clode, 2009; McCully et al., 2010; Marshall et al., 2012; Jin et al., 2017; Marshall, 2017; Hayes et al., 2018; Ondrasek et al., 2019). This approach avoided any loss or movement of elements and allowed for analysis of samples in a frozen-hydrated state, thus reflecting structure and cellular concentrations as they were at the time of harvest. Plunge-freezing directly into liquid N does result in relatively slow freezing and the potential for ice crystal formation. However, the method is still considered highly suitable for cell-level imaging and element analysis of plants, which are much more tolerant of slower freezing rates (see review by McCully et al., 2010). Methods such as high-pressure freezing, which improve freezing quality by reducing ice crystal formation, are limited to small (<200 μm) samples and are generally not suited to highly vacuolated samples (e.g., roots and leaves with large air spaces) due to the high pressures used during freezing. The use of liquid propane, on the other hand, could yield faster and improved freezing. However, due to high user risks, liquid propane becomes practically and operationally unfeasible. Areas of freezing damage evident in our samples are much smaller in scale than our regions of analysis, and in many cases, little-to-no damage is seen in plant samples frozen in liquid N at this scale (Ding et al., 2018; Guilherme Pereira et al., 2018; Hayes et al., 2019; Kotula et al., 2019; Ding et al., 2020). With this, we maintain that imaging and analysis of samples in their frozen state offers the best prospect of preserving cellular integrity, compared to chemical and resin-based preparations, which have the *potential* to extract components (e.g., lipids), create distortions (e.g., following dehydration), and result in the dissolution of ions (e.g., loss or movement of soluble ions and compounds).

## Conclusion

In conclusion, our examination of FRE using cryoSEM technology confirms differences between the morphological structures of FRE and those reported for AMF in the literature. Specifically, FRE possess uniformly thin hyphae compared with AMF, their colonization is restricted to the inner root cortex, and intercellular hyphae tend to occur in ropes and often form intercalary vesicle-like swellings. However, several similarities with AMF were found, hinting that FRE may respond to similar host-plant signals or that the host plant may employ a similar mechanism of association with FRE and AMF. Most importantly, this study provides evidence to support the hypothesis of FRE functionally acting as AMF at a cellular level. Previous studies conducted on non-vascular and early divergent plants (i.e., mycorrhizal-like symbioses) have suggested that FRE can transfer N and P to their hosts in exchange for carbon (Field et al., 2015, 2016, 2019; Hoysted et al., 2019); we now provide cytological evidence that such relationship also occurs in vascular plants that form true mycorrhizal symbioses. Further research is now required on the ecology and lifecycle of FRE to understand their function and interactions with glomeromycotinian AMF. Particularly, more studies comparing the nutritional roles of both groups of fungi in vascular plants, such as those of Field et al. (2015, 2016, 2019) conducted on liverworts, will be of great relevance.

## Data Availability Statement

The original contributions presented in the study are publicly available. This data can be found in NCBI, under accession number PRJNA639388.

## Author Contributions

FA, MR, and SO wrote the manuscript. FA conducted the statistical analyses. NN conducted the important preliminary experimental work. PH and PC obtained the cryoSEM images and X-ray elemental data. MR and RS provided the supervision of SO during her Ph.D. GB and SH conducted the all molecular and bioinformatics work. All authors contributed to the manuscript by providing feedback and/or written content.

## Conflict of Interest

The authors declare that the research was conducted in the absence of any commercial or financial relationships that could be construed as a potential conflict of interest.
