# Supplementary material for: First Cryo-Scanning Electron Microscopy Images and X-Ray Microanalyses of Mucoromycotinian Fine Root Endophytes in Vascular Plants
Source: Front Microbiol. 2020 Sep 3;11:2018. doi: 10.3389/fmicb.2020.02018 (PMC7509483; doi:10.3389/fmicb.2020.02018)
Supplement: Supplementary file 1 [file Presentation_1.pdf]

First cryo-scanning electron microscopy images and X-ray microanalyses of mucoromycotinian fine root endophytes in vascular plants

Felipe E. Albornoz, Patrick E. Hayes, Suzanne Orchard, Peta L. Clode, Nazanin K. Nazeri, Rachel J. Standish, Gary D. Bending, Sally Hilton, Megan H. Ryan

Supporting Information

The following Supporting information is available for this article:

Table S1 Taxonomy assigned to the nine fungal OTUs found in the roots studied.

Figure S1 Example qualitative element maps and spectra.

Figure S2 Cryo-scanning electron micrographs showing uncolonised cells

Table S1. Summary table of taxonomy assigned to the nine fungal OTUs found in the roots studied.

OUT ID	Sequences	Assigned taxonomy	Guild
OUT_1	3094	Fungi; Mucoromycota; Mucoromycotina; Incertae Sedis; Endogonales; Endogonaceae; uncultured	FRE
OUT_18	149	Fungi; Mucoromycota; Mucoromycotina; Incertae Sedis; Endogonales; Endogonaceae; uncultured	FRE
OUT_21	120	Fungi; Mucoromycota; Mucoromycotina; Incertae Sedis; Endogonales; Endogonaceae; uncultured	FRE
OUT_23	106	Fungi; Mucoromycota; Mucoromycotina; Incertae Sedis; Endogonales; Endogonaceae; uncultured	FRE
OUT_48	25	Fungi; Mucoromycota; Mucoromycotina; Incertae Sedis; Endogonales; Endogonaceae; uncultured	FRE
OUT_49	24	Fungi; Chytridiomycota; Incertae Sedis; Chytridiomycetes	other
OUT_52	21	Fungi; Mucoromycota; Mucoromycotina; Incertae Sedis; Endogonales; Endogonaceae; uncultured	FRE
OUT_79	4	Fungi; Cryptomycota; LKM11	other
OUT_84	3	Fungi; Basidiomycota; Pucciniomycotina; Microbotryomycetes; Sporidiobolales; Sporidiobolaceae; Rhodotorula	other

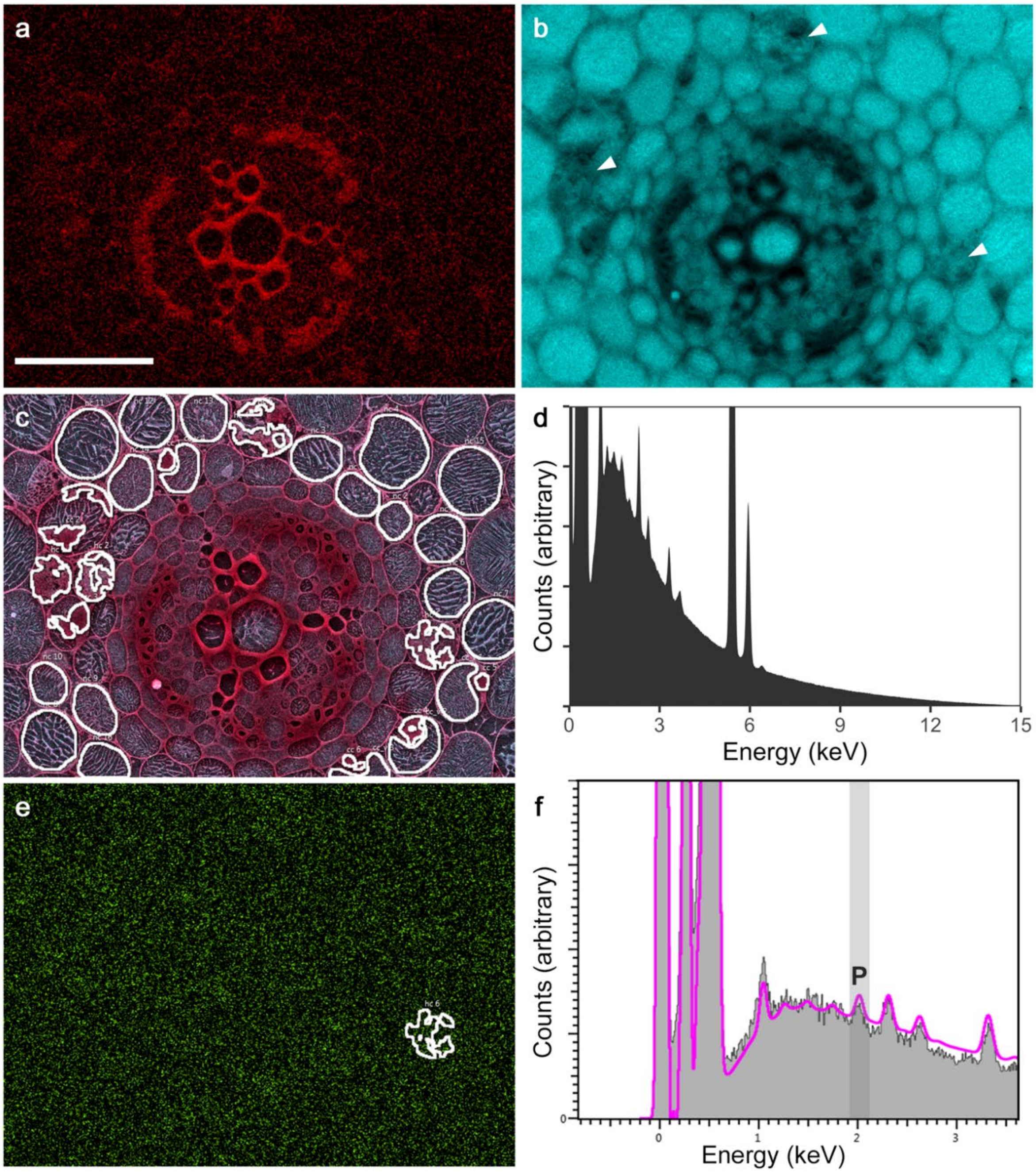

Figure S1. Qualitative element maps, layered electron micrograph, and corresponding spectra for a typical transverse *Trifolium subterraneum* root section, showing fine root endophyte (FRE) colonisation. (*a*-*c*) Matching qualitative element maps for C (*a*) and O (*b*) were layered onto the electron micrograph (*c*), revealing individual cells, sites of colonisation (arrowheads) and their corresponding highlighted regions of interest (ROI). Note, samples were in a frozen fully-hydrated state with slight etching used to reveal features. Specific ROI were identified using layered electron micrographs (*c*) and were drawn onto element maps using Oxford Instruments Aztec Energy software. Pixels within each ROI were combined and processed to yield quantitative element concentrations from individual cells and areas of interest. (*d*) Spectrum from the whole mapped region in *c*, showing that the sample was stable, with X-ray counts out to 15 keV. (e) Qualitative element map showing the distribution of P over the same mapped area, with a single ROI

Figure S1 (continued), showing a developed arbuscule. We did not present elemental maps in the main manuscript, because they did not clearly show the distribution of the main elements of interest, in particular P. This was due to consistently low concentrations. However, P and other elements were present and could be readily quantified from ROI, as evidenced by the example spectrum in *f*. (*f*) Extracted spectrum from the highlighted ROI (*e*), showing the raw spectrum from the ROI (grey) and the corrected and fitted spectrum used for quantitation (pink), with the P peak clearly identified (shaded region), even though the P concentration is low. This shows that although the distribution of P was not visually reflected in the qualitative maps, the P was present and readily quantifiable, as is true for all elements quantified in this study. Scale bar: *a-d,* 50 µm.

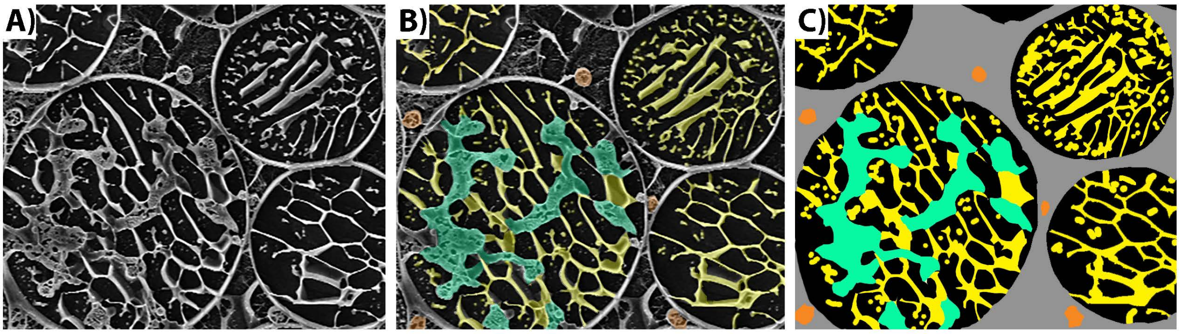

Figure S2, (A) Cryo-scanning electron micrographs of one single cells from the inner cortex colonized by fine root endophytes and three uncolonised cells of a root of *Trifolium subterraneum* in transverse section. (B) The same micrograph, now with key fungal structures highlighted in color: single intercellular hyphae (orange), developed arbuscule branches (green), and crystalized solutes (yellow). (C) Anatomical schematic showing only the structures of interest where gray and black represent intercellular and intracellular space, respectively.
